# Accelerating sow nursing behavior monitoring with modified YOLO11n architecture and TensorRT integration

**DOI:** 10.1186/s40813-026-00507-3

**Published:** 2026-04-22

**Authors:** Mamunur Rahman, Victor Hugo Silva Souza, Tami M. Brown-Brandl, Gary A. Rohrer, Yeyin Shi, Isabella C. F. S. Condotta

**Affiliations:** 1 Department of Animal Sciences, University of Illinois Urbana- Champaign, Urbana, IL USA; 2 Department of Biological Systems Engineering, University of Nebraska-Lincoln, Lincoln, NE USA; 3 USDA-ARS, US Meat Animal Research Center, Clay Center, NE USA

**Keywords:** Precision livestock farming, Computer vision, Deep learning, Preweaning mortality, Farrowing crates, Real-time monitoring, Inference acceleration

## Abstract

**Background:**

Efficient monitoring of sow nursing behavior is relevant to animal welfare monitoring and management of preweaning mortality (PWM) risk, which remains a significant challenge in intensive swine production systems. Traditional observation methods are labor-intensive and inadequate for large-scale farms, requiring automated solutions. Precision livestock farming (PLF) technologies, particularly computer vision and deep learning models, offer opportunities for continuous monitoring of sow posture–nursing-state classes. The YOLO (you only look once) architecture has demonstrated high accuracy and speed for object detection in different environments. This study developed and evaluated a modified YOLO11n model optimized with TensorRT to monitor sow posture–nursing-state classes in farrowing crates. In this study, “nursing” was operationalized from top-view images as visible piglet snout/mouth contact oriented toward the udder/teat line; frames without visually confirmable contact were labeled not nursing. The model’s lightweight architecture and inference acceleration address the computational constraints of real-time applications in farrowing-room monitoring.

**Results:**

The modified YOLO11n achieved an mAP@50 of 98.90%, while reducing complexity to 207 layers and 5.0 GFLOPs by removing the small-object detection head. This enabled inference times of 4.6 ms on an NVIDIA A100 GPU and 6.1 ms on a T4 GPU with TensorRT optimization. The framework detected and classified ten posture–nursing-state classes (sitting, standing, and three lying postures, each labeled as nursing or not nursing) on representative test images, including frames with partial occlusion and variable lighting. Misclassifications were observed primarily among visually similar classes, such as Sow_Sitting_Nursing and Sow_Sitting_Not_Nursing, consistent with limited visibility of piglet-to-teat contact in some postures from the top-view angle. TensorRT optimization further reduced inference latency by 58.56% (A100) and 44.55% (T4), demonstrating consistent inference-latency reductions on the evaluated GPU platforms.

**Conclusions:**

This study demonstrates the potential of leveraging lightweight deep learning architectures for real-time behavioral monitoring in PLF. The modified YOLO11n and TensorRT optimization provide an efficient framework for automated monitoring of sow posture–nursing-state classes, supporting welfare-oriented monitoring workflows relevant to PWM risk management. Future work will evaluate lightweight temporal post-processing and embedded deployment to reduce frame-to-frame label flicker and assess performance under on-farm compute and I/O constraints.

## Background

The growing global population is expected to surpass 9 billion by 2050, posing a significant challenge to food security. To satisfy the food requirements of the expanding population, agricultural production must increase by 70% [1, 2]. Pork, an essential source of animal protein, plays a critical role in addressing this demand because of its high feed conversion efficiency and broad acceptance [3]. Over the past five decades, genetics, nutrition, and husbandry advancements have substantially increased pork production [4, 5]. However, these achievements have introduced challenges, particularly in maintaining animal welfare and productivity during critical periods, such as the preweaning phase [6].

The preweaning period, typically 21–28 days, is crucial for piglet survival, growth, and immune development. During this stage, nursing behaviors are expressed through sow postural and piglet-contact patterns, and lateral lying positions often facilitate access to the udder/teat line [7, 8]. Postures such as sitting or standing can coincide with initiation or termination of piglet contact, and top-view visibility of snout-to-teat contact can be limited in these configurations, complicating consistent monitoring from overhead imagery. Disruptions to nursing caused by environmental stressors or sow health issues can lead to reduced weight gain, compromised immunity, and increased preweaning mortality (PWM), which has been reported to exceed 16% in intensive systems [9, 10]. PWM is an economic burden and raises ethical concerns, emphasizing the need for advanced monitoring systems for sow nursing behavior in farrowing crates [11–13].

Traditional methods of monitoring sow and piglet behaviors, such as manual observation, are labor-intensive, subjective, and increasingly impractical for modern large-scale operations [14, 15]. With the intensification of pig farming, automated, real-time monitoring solutions have become increasingly important [16]. Precision livestock farming (PLF) integrates advanced technologies such as computer vision and deep learning to support animal welfare and productivity [17, 18]. In particular, computer vision has proven effective for detecting and analyzing livestock behaviors under dynamic farm conditions [19–21].

Among deep learning models, the YOLO (you only look once) series has been widely adopted for real-time object detection because of its single-stage architecture, which combines bounding box prediction and class probability estimation [22–24]. The YOLO model family has gained considerable recognition for its speed and accuracy in object identification tasks [25, 26]. YOLO models are increasingly utilized in livestock management for posture recognition and behavior monitoring [27, 28]. The latest iteration, YOLO11, incorporates features such as the spatial pyramid pooling-fast (SPPF) module and convolutional block with parallel spatial attention (C2PSA) [29–31]. These architectural enhancements can improve feature extraction and performance under challenging conditions such as occlusion and variable lighting [32].

YOLO11n, a lightweight variant of YOLO11, is particularly suitable for edge applications because of its balance of computational efficiency and detection accuracy [33]. Combining TensorRT (NVIDIA’s deep learning inference acceleration library) with earlier YOLO models can improve processing speed while maintaining competitive accuracy. TensorRT employs techniques such as precision calibration and kernel fusion to increase inference speed, supporting real-time applications in resource-constrained environments [34–36].

Several studies have investigated automated assessment of sow posture and nursing-related behaviors in farrowing systems; however, practical deployment remains constrained by inference latency, hardware cost, and the challenge of defining nursing consistently from overhead imagery when piglet-to-udder contact is partially occluded [7, 8, 24, 28]. To address these constraints, this study focuses on a lightweight detector and hardware-level acceleration with minimal computational overhead while using an operational definition of nursing based on visually confirmed piglet engagement at the udder/teat line.

The primary objective of this study is to design a lightweight, computationally efficient model capable of accurately detecting and classifying sow posture–nursing-state in real time. For that, this study optimizes YOLO11n with TensorRT to improve inference speed for automated posture and nursing-state classification in farrowing crates, supporting deployment considerations for commercial swine production systems and provides automated posture–nursing-state monitoring that can support time-based management analytics, with welfare-relevant applications requiring farm-specific integration and validation.

## Methods

### Study site and animal housing

The study was conducted at USMARC in Clay Center, Nebraska. Behavioral imaging data were collected from five farrowing groups of Large White and Landrace sows. A farrowing group was defined as 20 sows and their litters housed concurrently in the same farrowing room.

The farrowing facility contained three farrowing rooms. Each room housed 20 individual farrowing stalls arranged in two rows of ten facing each other across a 1.2-m-wide central alley, with additional alleys located behind each row. Each stall included a sow feeder and nipple drinkers accessible to both the sow and piglets, and a designated creep area equipped with one or more heat lamp zones. Flooring consisted of slatted metal, and rubber mats were placed under the heat lamps in the creep areas. No bedding material was provided, and creep areas were not enclosed; the heat-lamp zone served as the primary localized warming area for piglets. The facility operated as a continuous-flow system, with new sows entering each farrowing room every 6 weeks.

Environmental control relied on hallway air that was conditioned before entering each room. During warm conditions, evaporative cooling in the central hallway cooled incoming air, which was distributed into the rooms through baffles at one end in a tunnel-ventilation configuration. During cold conditions, hallway air was heated using two forced-air heaters, and small wall vents running the length of each room served as air inlets to support air mixing during minimum ventilation. Each room also contained two overhead radiant heaters to provide supplemental heat. Lighting was maintained at 12 h/d (05:30 to 17:30) using 20 T-8 fluorescent bulbs.

Routine piglet husbandry practices followed USMARC protocols and included ear tagging and weighing at 1 day of age and processing (tail docking, teeth clipping, castration, and iron supplementation) at 3 days of age. Cross-fostering was implemented to equalize litter sizes during the first 3 days of life.

Images used in this manuscript originated from a separate experiment that evaluated farrowing stall layout and heat-lamp configuration within the same USMARC farrowing facility. Room-level characteristics were consistent across the facility (three rooms; 20 stalls/room; ventilation and lighting as described above), whereas stall-level dimensions and creep-area configurations varied by treatment. Three stall layouts were evaluated, and each layout was equipped with either one heat lamp (1HL) or two heat lamps (2HL) in a 2 × 3 randomized block design, with blocking by farrowing crate location relative to the ventilation fans. The Traditional Stall Layout (TSL) measured 1.52 × 2.13 m overall with a central sow crate measuring 0.61 × 2.13 m. The Expanded Creep Area Stall Layout (ECSL) retained the 0.61 × 2.13 m sow crate and increased total stall area to 1.83 × 2.44 m. The Expanded Sow and Creep Area Stall Layout (ESCSL) measured 1.83 × 2.44 m overall with a sow crate measuring 0.71 × 2.13 m. Sows were moved into farrowing stalls approximately 5 d before the expected farrowing date to support routine facility management and acclimation. For the present manuscript, analyses used a subset of images drawn from this larger experiment and the modeled dataset was restricted to images collected from day 0 (farrowing) through weaning (21 days after farrowing) of 5 farrowing groups; stall-layout metadata were retained to characterize the housing context of the annotated images.

### Data acquisition

The recording system was deployed across the three farrowing rooms (20 stalls/room; 60 stalls total). Above each row of stalls, a 21.6-m aluminum theatre triangle truss was installed at approximately 2.6 m above the floor. A Kinect V2^®^ sensor was positioned above each stall (20 sensors/room). Only the RGB stream was used in this study, with a resolution of 1920 × 1080 pixels. Sensors were mounted to minimize occlusions and to maintain consistent top-view coverage of sow posture and piglet location within each stall. System checks and recalibration were performed as needed to maintain stable image quality and spatial alignment over time.

Data capture and storage were managed through a network of mini-PCs, with recordings stored on a RAID-configured network-attached storage system (Synology DS1517 + NAS; 50 TB total capacity). RGB images were sampled at 0.2 frames/s (one image every 5 s) and collected continuously for 22 d per farrowing group (birth to weaning). Files were organized by farrowing group, sow ID, and date to support retrieval and downstream processing. Backups consisted of daily incremental and weekly full backups, with a secondary copy stored offsite.

### Behavioral annotation

**Fig. 1:**
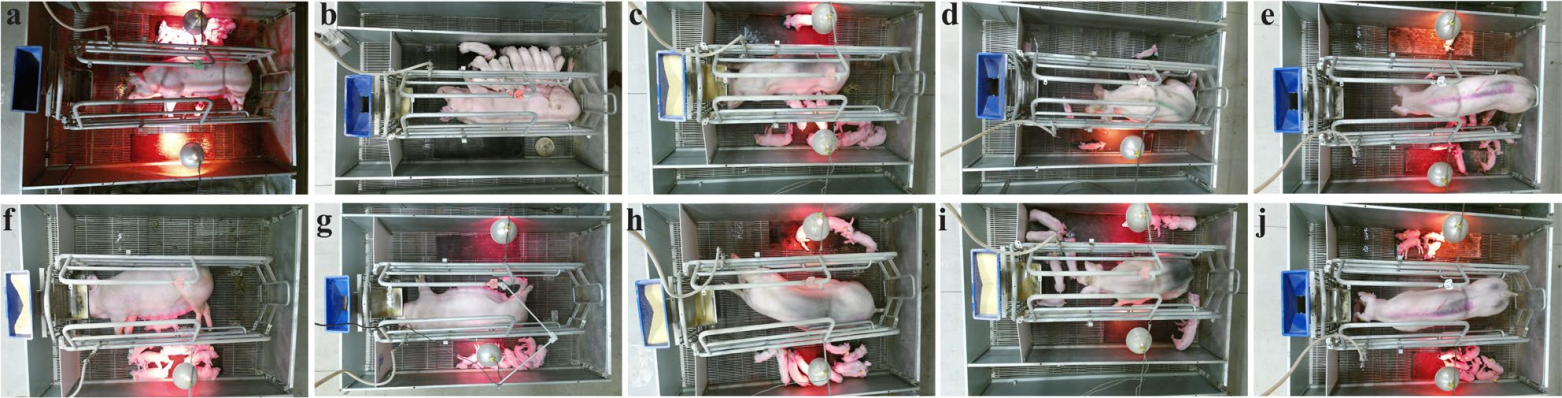
Images of ten sow posture–nursing-state classes in farrowing stalls, classified as nursing or not nursing. Panels a–e show nursing-labeled postures (based on the operational definition): (**a**) Sow_Lying_Right_Nursing, (**b**) Sow_Lying_Left_Nursing, (**c**) Sow_Sternal_Lying_Nursing, (**d**) Sow_Sitting_Nursing, and (**e**) Sow_Standing_Nursing. Panels f–j show not-nursing-labeled postures: (**f**) Sow_Lying_Right_Not_Nursing, (**g**) Sow_Lying_Left_Not_Nursing, (**h**) Sow_Sternal_Lying_Not_Nursing, (**i**) Sow_Sitting_Not_Nursing, and (**j**) Sow_Standing_Not_Nursing. Nursing labels required visible snout/mouth contact at the udder/teat line; frames where contact could not be visually confirmed were labeled not nursing

A total of 5,000 images were selected for annotation with approximately balanced representation across 10 posture–nursing-state classes derived from the combination of two behavioral states (nursing, not nursing) and five sow postures (lateral lying left, lateral lying right, sternal lying, sitting, standing). Candidate images were curated from the continuous 0.2 frames/s image stream using a stratified approach to achieve approximately balanced representation across the 10 posture–nursing-state classes while reducing redundancy from temporally adjacent frames. Curation was performed in CVAT using separate tasks per class, where the annotator reviewed candidate frames and preferentially retained visually distinct examples while skipping near-duplicate consecutive frames from the same sow. To increase within-class variation, higher-occurrence lying classes were down-sampled more aggressively by skipping larger runs of temporally adjacent frames from the same sow, whereas rarer sitting and standing classes were curated with shorter spacing (typically retaining examples separated by at least 10–15 frames when consecutive candidates were available). Frames with insufficient visibility for applying the ethogram (e.g., severe motion blur, incomplete sow body in frame, or lighting artifacts that prevented assessment of piglet snout-to-udder/teat-line contact) were excluded prior to annotation. Images were annotated in the open-source Computer Vision Annotation Tool (CVAT) and assigned to one of the 10 classes: Sow_Lying_Right_Nursing, Sow_Lying_Left_Nursing, Sow_Sternal_Lying_Nursing, Sow_Sitting_Nursing, Sow_Standing_Nursing, Sow_Lying_Right_Not_Nursing, Sow_Lying_Left_Not_Nursing, Sow_Sternal_Lying_Not_Nursing, Sow_Sitting_Not_Nursing, and Sow_Standing_Not_Nursing (Fig. 1). Each annotation included a bounding box enclosing the sow and the corresponding class label.

An ethogram was used to standardize labeling from top-view images (Table 1). Nursing-state versus not-nursing-state was determined based on visible piglet engagement at the udder/teat line (snout/mouth contact oriented toward the udder region). This operational definition identifies frames with visually confirmed piglet udder/teat-line engagement, which can occur during different phases of a nursing bout, but it does not confirm milk letdown or the sow’s nursing “call.” Frames in which contact could not be visually confirmed were labeled as not nursing.

**Table 1:** Ethogram and operational definitions used to label sow posture–nursing-state classes from the top-view images

Posture class (abbrev.)	Operational definition
Sow_Lying_Left_Nursing (S_LL_N)	Sow is lying flat on its left side; ≥ 1 piglet at the udder/teat line with snout/mouth visibly contacting the teat/udder (head oriented toward the udder).
Sow_Lying_Left_Not_Nursing (S_LL_XN)	Sow is lying flat on its left side; no piglet visibly engaged at the udder/teat line (no visible snout-to-teat region contact).
Sow_Lying_Right_Nursing (S_LR_N)	Sow is lying flat on its right side; ≥1 piglet at the udder/teat line with snout/mouth visibly contacting the teat/udder (head oriented toward the udder).
Sow_Lying_Right_Not_Nursing (S_LR_XN)	Sow is lying flat on its right side; no piglet visibly engaged at the udder/teat line (no visible snout-to-teat region contact).
Sow_Sternal_Lying_Nursing (S_SL_N)	Sow in sternal recumbency (sternum/chest on floor); ≥ 1 piglet at the udder/teat line with snout/mouth visibly contacting the teat/udder (head oriented toward the udder).
Sow_Sternal_Lying_Not_Nursing (S_SL_XN)	Sow in sternal recumbency (sternum/chest on floor); no piglet visibly engaged at the udder/teat line (no visible snout-to-teat region contact).
Sow_Sitting_Nursing (S_Si_N)	Sow sitting (hindquarters on floor, trunk upright); ≥ 1 piglet at the udder/teat line with snout/mouth visibly contacting the teat/udder (head oriented toward the udder).
Sow_Sitting_Not_Nursing (S_Si_XN)	Sow sitting (hindquarters on floor, trunk upright); no piglet visibly engaged at the udder/teat line (no visible snout-to-teat region contact).
Sow_Standing_Nursing (S_St_N)	Sow standing on all four limbs; ≥ 1 piglet at the udder/teat line with snout/mouth visibly contacting the teat/udder (head oriented toward the udder).
Sow_Standing_Not_Nursing (S_St_XN)	Sow standing on all four limbs; no piglet visibly engaged at the udder/teat line (no visible snout-to-teat region contact).

The annotated dataset was split into training and validation sets at an 80:20 ratio with stratification by class to preserve class balance in each subset. The split was performed at the image level; therefore, images from the same sow could occur in both subsets. The curation rules above described were applied to reduce near-duplicate consecutive frames and limit temporal autocorrelation between subsets. Accordingly, validation performance should be interpreted as within-facility performance rather than fully independent generalization to unseen animals or facilities.

To quantify labeling consistency, two trained annotators independently labeled a stratified subset of 300 images (30 per class) using the same ethogram and CVAT interface. Overall percent agreement was 96.67% (290/300). Chance-corrected agreement was quantified using Cohen’s kappa, computed as:

$$\:\kappa\:=\frac{{P}_{o}-{P}_{e}}{1-{P}_{e}},$$

where $\:{P}_{o}$ is the observed proportion of agreement and $\:{P}_{e}$ is the expected agreement by chance based on the marginal label distributions. In this stratified sample with 10 classes represented equally, $\:{P}_{o}=0.9667$and $\:{P}_{e}=0.10$, yielding $\:\kappa\:=0.963$. Per-class agreement ranged from 90.0% to 100.0%, with the lowest agreement observed for the sitting posture–nursing-state classes (Sow_Sitting_Nursing vs. Sow_Sitting_Not_Nursing; Table 2). Discrepancies were resolved by consensus prior to finalizing the training labels.

**Table 2:** Inter-observer agreement for sow posture–nursing-state class labels based on independently annotated subset of images (*N* = 300; 30 images per class)

Posture–nursing-state classes (abbrev.)	Images (*n*)	Agreement (*n*)	Disagreement (*n*)	Agreement (%)	Most common disagreement (if any)
Sow_Lying_Left_Not_Nursing (S_LL_XN)	30	30	0	100.0	
Sow_Lying_Left_Nursing (S_LL_N)	30	29	1	96.7	S_LL_N ↔ S_LL_XN
Sow_Lying_Right_Not_Nursing (S_LR_XN)	30	30	0	100.0	
Sow_Lying_Right_Nursing (S_LR_N)	30	29	1	96.7	S_LR_N ↔ S_LR_XN
Sow_Sitting_Not_Nursing (S_Si_XN)	30	27	3	90.0	S_Si_XN ↔ S_Si_N
Sow_Sitting_Nursing (S_Si_N)	30	27	3	90.0	S_Si_N ↔ S_Si_XN
Sow_Standing_Not_Nursing (S_St_XN)	30	29	1	96.7	S_St_XN ↔ S_St_N
Sow_Standing_Nursing (S_St_N)	30	30	0	100.0	
Sow_Sternal_Lying_Not_Nursing (S_SL_XN)	30	29	1	96.7	S_SL_XN ↔ S_SL_N
Sow_Sternal_Lying_Nursing (S_SL_N)	30	30	0	100.0	
**Overall**	**300**	**290**	**10**	**96.67**	

### Data augmentation and preprocessing

Data augmentation was applied during training to increase robustness to variation in lighting, occlusion, and viewpoint within stalls. Augmentations included mosaic (*p* = 1.0), RandAugment, and random erasing (*p* = 0.4). Geometric transformations included random scaling (scale = 0.5), translation (fraction = 0.1), and rotation (± 5°). Photometric augmentation included HSV jitter (hue ± 0.015; saturation ± 0.7; value ± 0.4).

### YOLO11 architecture and modifications

**Fig. 2:**
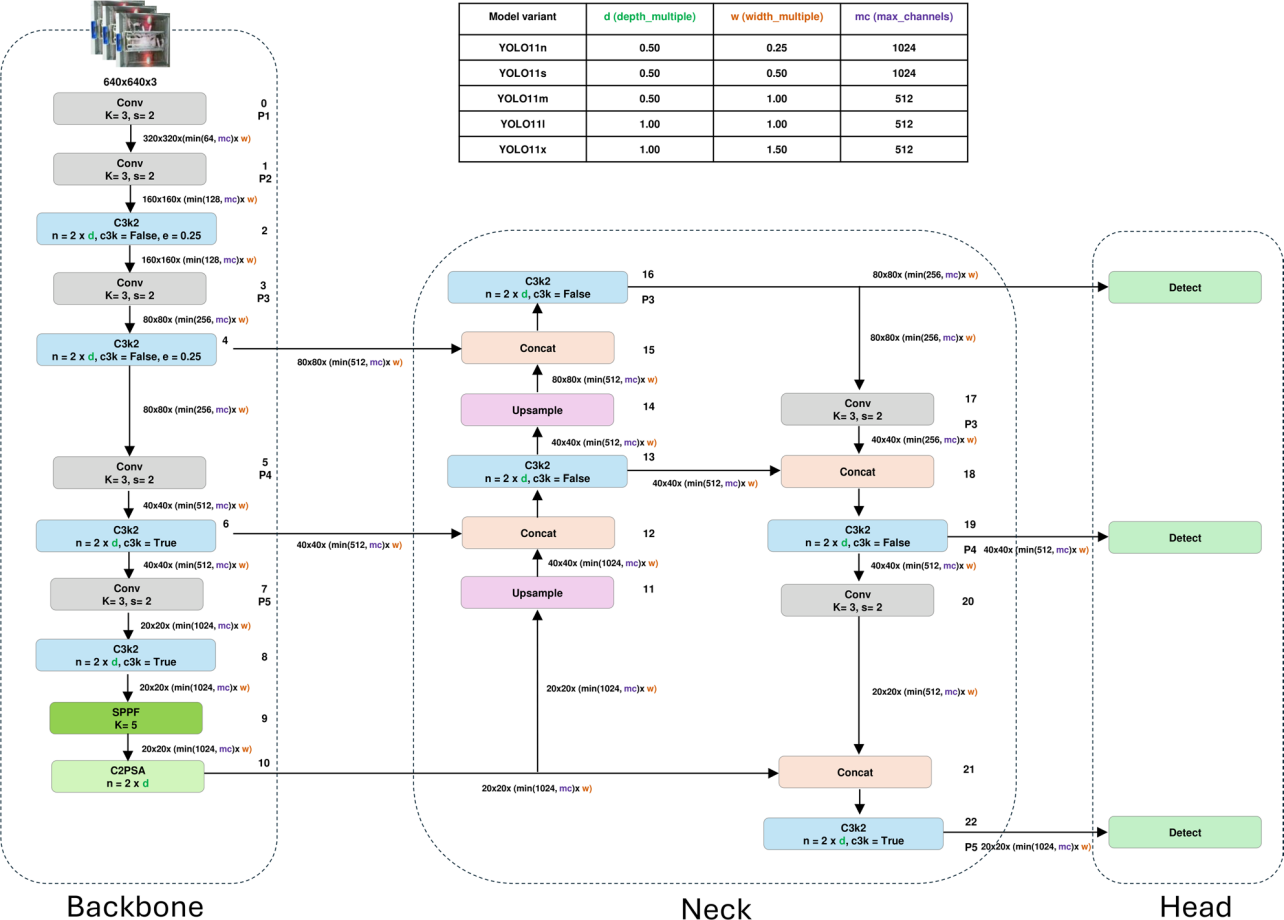
General architecture and key configurations of YOLO11 model variants. The YOLO11 architecture comprises three components: a backbone for feature extraction via C3k2 blocks (k = 2, kernel size) and spatial pyramid pooling-fast (SPPF); a neck for aggregating features by upsampling, concatenation, and additional C3k2 blocks (n, number of bottleneck blocks); and a head for predicting bounding boxes and class probabilities optimized for medium and large objects. The accompanying table summarizes key parameters: depth (d), width (w), and maximum number of channels (mc) for YOLO11 variants

The YOLO11 architecture (Fig. 2) comprises three main components: Backbone, Neck, and Head, each designed for object detection and adapted here for classification of sow posture–nursing-state classes. These components collaborate to facilitate efficient and precise detection of sow posture–nursing-state classes in farrowing crates.

#### Backbone

The backbone is the foundational component for extracting hierarchical features from input images. This is achieved through a series of convolutional layers that progressively transform raw image data into multiscale feature maps. YOLO11 uses C3k2 blocks, an optimized version of the cross-stage partial (CSP) bottleneck characterized by a smaller kernel size (k = 2). Reducing the kernel size improves computational efficiency by reducing the processing time while maintaining the ability to capture essential image features.

Another critical addition to the backbone is the spatial pyramid pooling-fast (SPPF) module. This module pools spatial information from multiple image regions and combines it into a unified feature map, enabling detection of objects even under challenging conditions.

#### Neck

The neck serves as an intermediate processing stage that aggregates multiscale features generated by the backbone. These features are refined and prepared for detection in the head. YOLO11 incorporates additional C3k2 blocks in the neck to streamline the aggregation of feature maps while minimizing computational complexity.

To further enhance feature processing, the convolutional block with parallel spatial attention (C2PSA) module is integrated into the neck. This module employs spatial attention mechanisms to focus the model’s processing power on the most relevant regions of the image, such as areas where sows are labeled as nursing or changing postures. This enhancement improves the model’s ability to detect objects of varying sizes. This design can help maintain performance even when sows are partially occluded or positioned at the edges of the farrowing crate.

#### Head

The head is the final component responsible for generating predictions, including bounding boxes, class probabilities, and confidence scores for detected objects. YOLO11 includes three detection heads, each optimized for detecting small, medium, and large objects. These detection layers process the refined multiscale features from the neck and generate precise predictions, supporting reliable detection and monitoring objects.

#### Architecture Modifications for YOLO11n

**Fig. 3:**
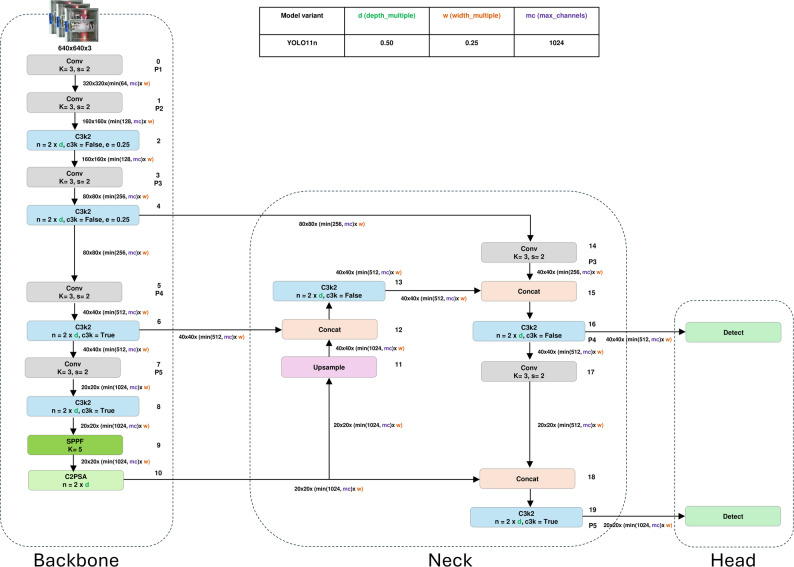
Modified YOLO11n architecture with reduced detection heads. The modified YOLO11n architecture retains the backbone, neck, and two detection heads optimized for medium and large objects. The small-object detection head has been removed to increase computational efficiency and reduce complexity while maintaining high accuracy for detecting sow posture-nursing-state

To adapt YOLO11 for efficient real-time applications, the lightweight variant YOLO11n was selected for further optimization. Following an evaluation of all five YOLO11 model variants (YOLO11n, YOLO11 s, YOLO11m, YOLO11l, and YOLO11x), modifications were made to the YOLO11n architecture to specifically address the computational constraints and focus on the detection of medium and large objects (Fig. 3). The small-object detection head and its associated layers were removed, as the primary goal was to detect medium and large objects such as sows. Eliminating small-object detection head does not alter the nursing vs. not nursing labeling rule. The model does not explicitly detect piglets as a separate object class; instead, the nursing versus not-nursing-state is learned from visual cues within the sow bounding box (e.g., visible piglet positioning and snout-to-udder/teat-line engagement) together with sow posture features. The model predicts ten sow posture–nursing-state classes, and the nursing-state is learned from the visual features (including piglet presence), not from sow posture alone.

This modification reduced the computational complexity during inference while retaining strong detection performance for the target object sizes. Focusing on medium- and large-object detection streamlined the architecture and reduced inference latency on the evaluated hardware, making it suitable for sow posture and nursing-label monitoring in farrowing crates. These tailored changes balance computational efficiency and detection accuracy, supporting a practical solution for real-time livestock monitoring tasks.

### TensorRT optimization

Further performance improvements were achieved via NVIDIA TensorRT, a deep learning inference optimization framework designed to increase deployment efficiency. TensorRT was applied to all six YOLO11 model variants, including YOLO11n, YOLO11 s, YOLO11m, YOLO11l, YOLO11x, and the modified YOLO11n. This framework incorporates advanced techniques such as kernel fusion, which merges multiple computational operations into a single kernel to reduce memory access and improve processing speed; dynamic tensor memory allocation, which optimizes resource utilization by allocating memory dynamically during inference; and mixed precision calibration, which uses lower precision (e.g., FP16 or INT8) calculations to achieve a balance between accuracy and computational efficiency. These optimizations collectively reduce inference latency, enabling real-time object detection while maintaining high performance. TensorRT’s flexibility supports deployment across the evaluated GPU platforms, from a high-performance GPU such as the NVIDIA A100 to a more resource-constrained system like the NVIDIA T4. TensorRT acceleration improved inference speed and efficiency for the evaluated YOLO11 variants, supporting low-latency livestock monitoring use cases where timely detection and intervention matter.

## Results

### Model performance

The performance metrics of the YOLO11 model variants are summarized in Table 3. Among the six variants, the modified YOLO11n achieved a comparable mAP@50 score (98.90%) to the larger models YOLO11 × (98.79%) and YOLO11l (98.85%) while requiring fewer parameters and fewer FLOPs. These results indicate that removing the small-object detection head did not materially affect mAP@50 for this task and dataset.

In terms of mAP@50:95, the modified YOLO11n achieved a value of 90.80%, which is lower than the larger YOLO11 × (94.23%) and YOLO11l (94.38%) values. These differences reflect trade-offs between model capacity and detection performance across stricter IoU thresholds. Nevertheless, the modified YOLO11n retained competitive mAP@50:95 with the smallest model footprint (2.55 M parameters; 5.0 GFLOPs).

**Table 3:** Performance metrics of YOLO11 model variants, including the modified YOLO11n

Model variant	Parameters	Layers	Precision (*P*)	Recall (*R*)	mAP@50 (%)	mAP@50:95 (%)	Validation inference speed (ms)	FLOPs	Training time (h)
YOLO11n	2,584,102	238	98.36%	98.27%	98.88%	93.71%	1.9	6.3	1.617
YOLO11s	9,416,670	238	98.33%	98.56%	98.87%	94.64%	2.2	21.3	1.619
YOLO11m	20,037,742	303	98.04%	98.17%	98.82%	94.38%	2.9	67.7	2.051
YOLO11l	25,287,022	464	97.73%	98.33%	98.85%	94.38%	3.6	86.6	2.617
YOLO11x	56,838,574	464	98.03%	98.20%	98.79%	94.23%	4.9	194.5	3.091
YOLO11n_Modified	2,552,748	207	98.04%	98.20%	98.90%	90.80%	1.6	5.0	1.493

The modified YOLO11n also demonstrated the fastest inference speed of 1.6 ms, outperforming larger models such as YOLO11 × (4.9 ms) and YOLO11l (3.6 ms). This reduction in latency, combined with its high mAP@50 performance, supports the modified YOLO11n as a practical choice for real-time applications in PLF. Because the “Validation Inference Speed (ms)” values in Table 3 reflect inference timing under the model validation setting used to produce those metrics, end-to-end timing (pre-inference + inference + post-inference) is reported separately in Table 4.

The proposed YOLO11n_modified has 207 layers instead of 238 layers in the original. This reduced computation from 6.3 GFLOPs to 5.0 GFLOPs and reduced training time from 1.617 h to 1.493 h, while maintaining similar precision (98.04%) and recall (98.20%) on the validation set.

### Loss function analysis

**Fig. 4:**
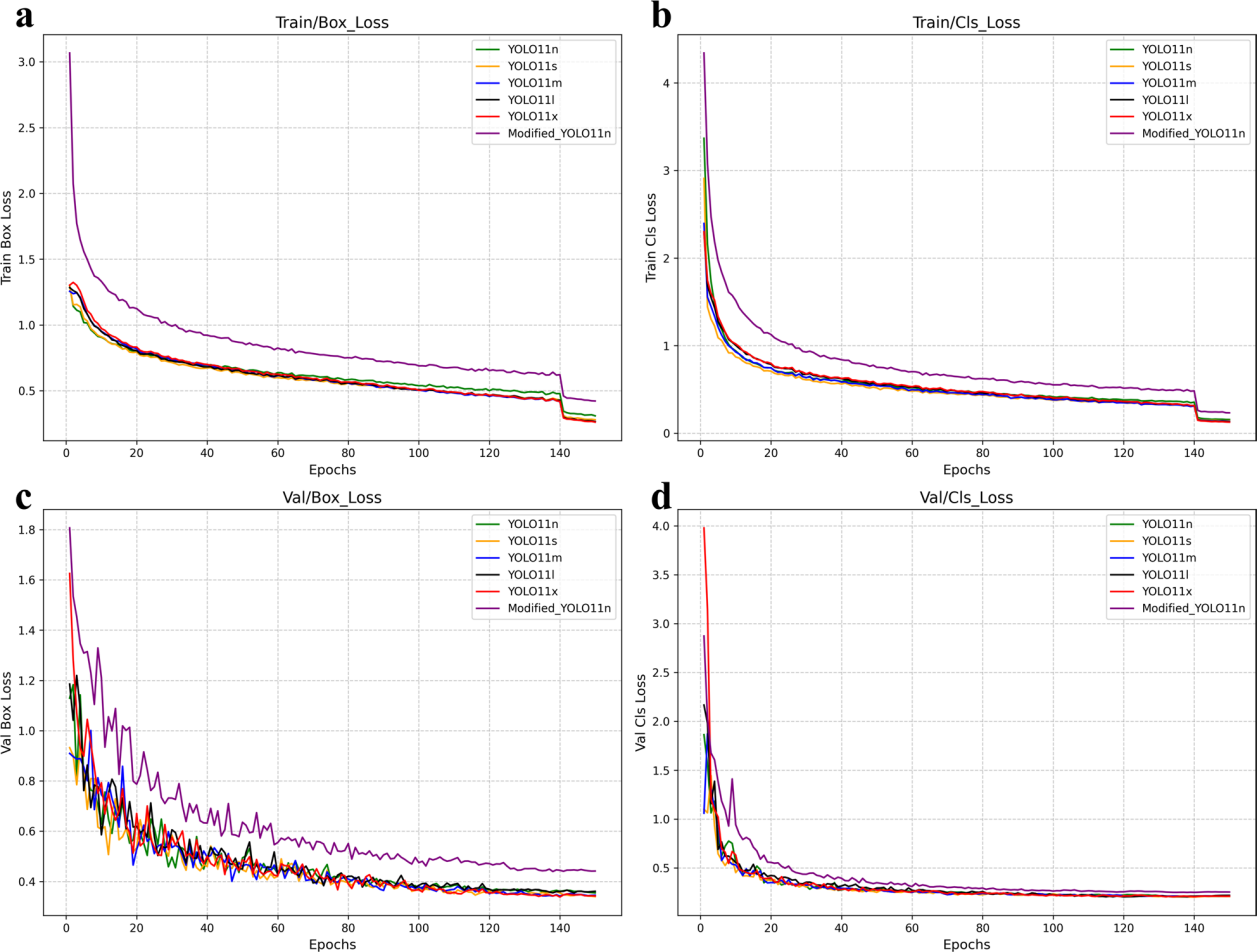
Training and validation loss trends for YOLO11 model variants over 150 epochs. (**a**) and (**b**) show the reduction in training box loss and classification loss, respectively, whereas (**c**) and (**d**) illustrate the validation box and classification loss, respectively

The training and validation loss trends for all YOLO11 variants are shown in Fig. 4. These graphs illustrate the reduction in box loss and classification loss across 150 epochs, indicating optimization patterns and architectural trade-offs. The training box loss (Fig. 4a) and training classification loss (Fig. 4b) exhibited consistent reductions across all YOLO11 variants, stabilizing after approximately 100 epochs. Among these, YOLO11n_modified consistently presented higher loss values than the other models did, consistent with reduced model capacity after removing the small-object detection head. The results indicate that while the YOLO11n_modified variant achieves computational efficiency, it sacrifices some optimization capability during training.

The larger models, such as YOLO11l and YOLO11x, demonstrated faster convergence and lower final loss values, consistent with reduced training loss for localization and classification. Notably, YOLO11s and YOLO11n maintained a trajectory comparable to those of these larger models, showing comparable convergence trends despite having fewer parameters. The validation box loss (Fig. 4c) and validation classification loss (Fig. 4d) showed similar patterns of steady reduction, with all the models converging toward minimal loss by the end of training. YOLO11n_modified exhibited higher validation loss values than the other models before stability was achieved, particularly in the early epochs. This divergence indicates reduced parameter optimization in the modified architecture, aligning with its design focus on computational efficiency over deep feature representation. In contrast, YOLO11x and YOLO11l maintained lower validation losses throughout training, which is consistent with their higher mAP@50:95 values on the validation set. YOLO11m, YOLO11s, and YOLO11n also showed consistent convergence patterns, with validation loss values approaching those of the larger models by the final epochs.

### Confusion matrix

**Fig. 5:**
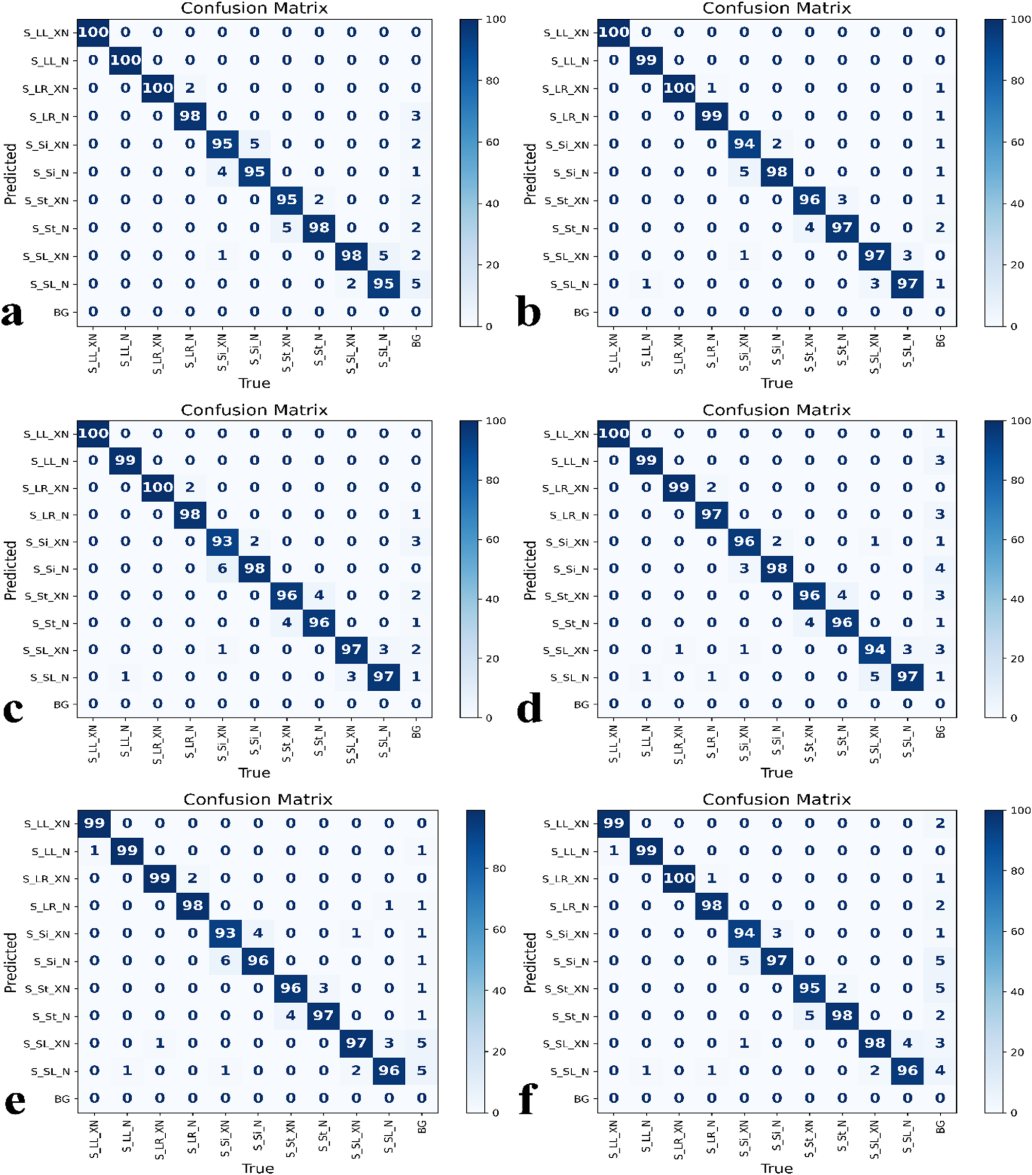
Confusion matrix comparison of YOLO11 model variants for classification of ten sow posture–nursing-state classes and a background (BG) class: (**a**) YOLO11n, (**b**) YOLO11s, (**c**) YOLO11m, (**d**) YOLO11l, (**e**) YOLO11x, and (**f**) YOLO11n_modified. Abbreviations: S_LL_XN (Sow_Lying_Left_Not_Nursing), S_LL_N (Sow_Lying_Left_Nursing), S_LR_XN (Sow_Lying_Right_Not_Nursing), S_LR_N (Sow_Lying_Right_Nursing), S_Si_XN (Sow_Sitting_Not_Nursing), S_Si_N (Sow_Sitting_Nursing), S_St_XN (Sow_Standing_Not_Nursing), S_St_N (Sow_Standing_Nursing), S_SL_XN (Sow_Sternal_Lying_Not_Nursing), and S_SL_N (Sow_Sternal_Lying_Nursing), BG (background)

The performance of the YOLO11 model variants was further evaluated using confusion matrices (Fig. 5), which summarize classification accuracy and error structure across the ten posture–nursing-state classes and a background (BG) class. Across models, misclassifications were concentrated within posture-matched nursing-labeled versus not-nursing-labeled pairs, consistent with the subtle visual differences between these states in top-view imagery.

For YOLO11n_modified (Fig. 5f), the dominant confusion occurred between Sow_Sitting_Nursing (S_Si_N) and Sow_Sitting_Not_Nursing (S_Si_XN), with 3 true S_Si_N images predicted as S_Si_XN and 5 true S_Si_XN images predicted as S_Si_N. A second common confusion was observed between Sow_Sternal_Lying_Nursing (S_SL_N) and Sow_Sternal_Lying_Not_Nursing (S_SL_XN), with 4 true S_SL_N images predicted as S_SL_XN and 2 true S_SL_XN images predicted as S_SL_N. Smaller, posture-matched confusion was also present for the standing pair (S_St_N → S_St_XN: 2; S_St_XN → S_St_N: 5). Misclassification between lateral-lying nursing-labeled versus not-nursing-labeled pairs was rare (S_LL_XN → S_LL_N: 1; S_LR_N → S_LR_XN: 1), and no consistent cross-posture confusion pattern was observed. Non-zero BG entries were ≤ 5% across classes, indicating infrequent missed detections under the evaluation setting used to generate Fig. 5.

This structure was broadly consistent across the other YOLO11 variants (Fig. 5a–e): errors were most frequently associated with the sitting pair and, depending on the model, the sternal-lying pair. For example, the sitting pair showed persistent bidirectional confusion across models (e.g., S_Si_N → S_Si_XN ranged from 2 to 5; S_Si_XN → S_Si_N ranged from 3 to 6), indicating that these posture–nursing-state classes represent the most challenging separation under the current imaging viewpoint and operational definition of nursing.

### Precision‒recall curve analysis

**Fig. 6:**
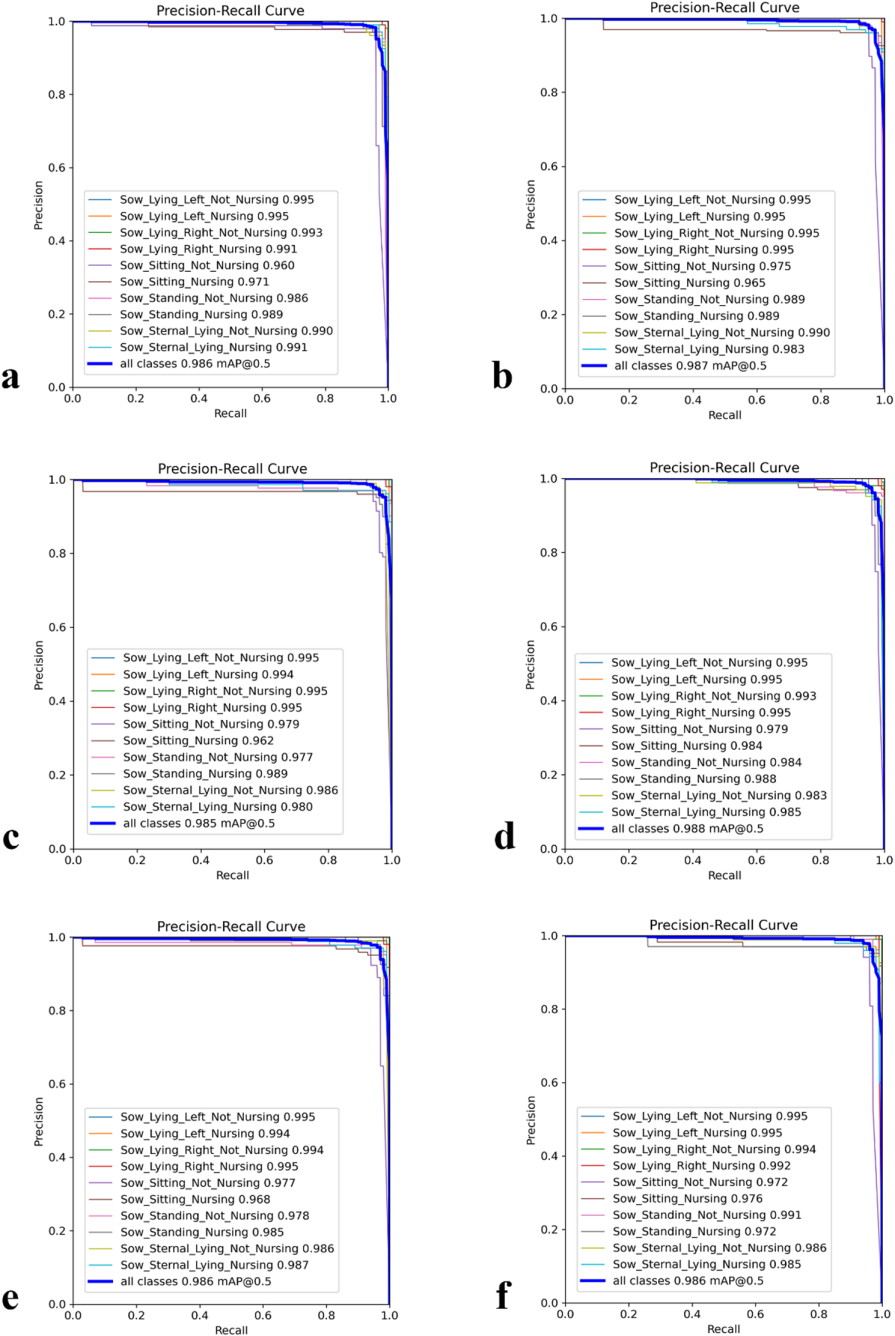
Precision-recall curves of six YOLO11 model variants for sow labeled nursing-state. (**a**) YOLO11n, (**b**) YOLO11s, (**c**) YOLO11m, (**d**) YOLO11l, (**e**) YOLO11x, and (**f**) modified YOLO11n. These curves evaluate classification performance across ten sow posture–nursing-state classes, illustrating precision and recall trade-offs for each class

The precision‒recall (PR) curves (Fig. 6) illustrate the performance of six YOLO11 variants (YOLO11n, YOLO11n_modified, YOLO11s, YOLO11m, YOLO11l, and YOLO11x) across the ten posture–nursing-state classes. All the models achieved high mAP@0.5 values, confirming strong multiclass performance at IoU = 0.5. Across variants, class-wise average precision (AP) values were highest for lateral-lying classes (typically ~ 0.99–0.995) and lowest for the sitting classes, particularly Sow_Sitting_Not_Nursing (as low as ~ 0.96 depending on the variant), consistent with the confusion-matrix structure.

### TensorRT optimization with YOLO11

Table 4 provides a comparative analysis of the inference performance for six YOLO11 model variants (YOLO11n, YOLO11s, YOLO11m, YOLO11l, YOLO11x, and YOLO11n_Modified) across two GPU platforms, NVIDIA A100 and T4, with and without TensorRT optimization. The table includes metrics such as pre-inference time, inference time, post-inference time, total inference time, and TensorRT speedup percentages, offering insights into computational efficiency.

**Table 4:** Inference performance comparison of YOLO11 variants with and without TensorRT optimization on NVIDIA A100 and T4 GPUs

Model variant	GPU	TensorRT	Pre-inference time (ms)	Inference time (ms)	Post-inference time (ms)	Total time (ms)	TensorRT speedup (%)
YOLO11n	A100	No	2	9.8	1.5	13.3	
	A100	Yes	2.1	1.5	2.4	6	54.89%
	T4	No	3	15.6	2.4	21	
	T4	Yes	1.7	3.5	1.5	6.7	68.1%
YOLO11s	A100	No	2.2	10.1	1.9	14.2	
	A100	Yes	1.8	1.8	1.5	5.1	64.08%
	T4	No	3.5	15.7	1.7	20.9	
	T4	Yes	1.8	5.4	1.5	8.7	58.37%
YOLO11m	A100	No	2.3	11.9	1.9	16.1	
	A100	Yes	1.8	2.4	1.6	5.8	63.98%
	T4	No	6.1	37	3.8	46.9	
	T4	Yes	2.1	10	1.9	14	70.15%
YOLO11l	A100	No	2.8	19.3	2.4	24.5	
	A100	Yes	2.8	3.3	1.5	7.6	68.98%
	T4	No	5.1	46.5	3.6	55.2	
	T4	Yes	2.7	12.9	1.6	17.2	68.84%
YOLO11x	A100	No	2.2	19	2	23.2	
	A100	Yes	1.8	4.4	1.6	7.8	66.38%
	T4	No	3.1	79.2	2.4	84.7	
	T4	Yes	2.8	22.2	2.4	27.4	67.65%
YOLO11n_modified	A100	No	1.7	8.1	1.3	**11.1**	
	A100	Yes	1.8	1.3	1.5	**4.6**	58.56%
	T4	No	2.6	7.2	1.2	**11**	
	T4	Yes	1.7	3	1.4	**6.1**	44.55%

Without TensorRT optimization, YOLO11n_modified demonstrated the fastest total inference time across both GPU platforms, achieving 11.1 ms on A100 and 11 ms on T4. Among the standard YOLO11 models, YOLO11n followed closely, with total inference times of 13.3 ms (A100) and 21 ms (T4). Larger models, such as YOLO11x and YOLO11l, require significantly greater inference times due to their increased complexity and parameter counts, with YOLO11x reaching 23.2 ms on A100 and 84.7 ms on T4, indicating their suitability for offline or batch processing rather than real-time applications.

TensorRT optimization significantly increased the inference speed for all YOLO11 variants. The magnitude of speedup varied by model and GPU platform (e.g., YOLO11n and YOLO11m showed larger relative gains on T4 than on A100, whereas YOLO11n_modified and YOLO11s showed larger gains on A100). After optimization, the YOLO11n_modified model exhibited the fastest inference times across both platforms, achieving 4.6 ms on A100 and 6.1 ms on T4, with speedup percentages of 58.56% and 44.55%, respectively. Similarly, YOLO11n reduced its total inference time on T4 from 21 ms to 6.7 ms, achieving a speedup of 68.1%.

Larger models, such as YOLO11x and YOLO11l, demonstrated considerable speed improvements with TensorRT optimization. YOLO11x reduced its total inference time by 66.38% on A100 and 67.65% on T4. However, its absolute inference times of 7.8 ms (A100) and 27.4 ms (T4) remained slower than those of the smaller YOLO11n_modified variant, highlighting the trade-off between accuracy and computational efficiency. YOLO11l achieved speedups of approximately 69% on both platforms, whereas YOLO11m exceeded 70% on T4 but was approximately 64% on A100.

### Predictions on test images

**Fig. 7:**
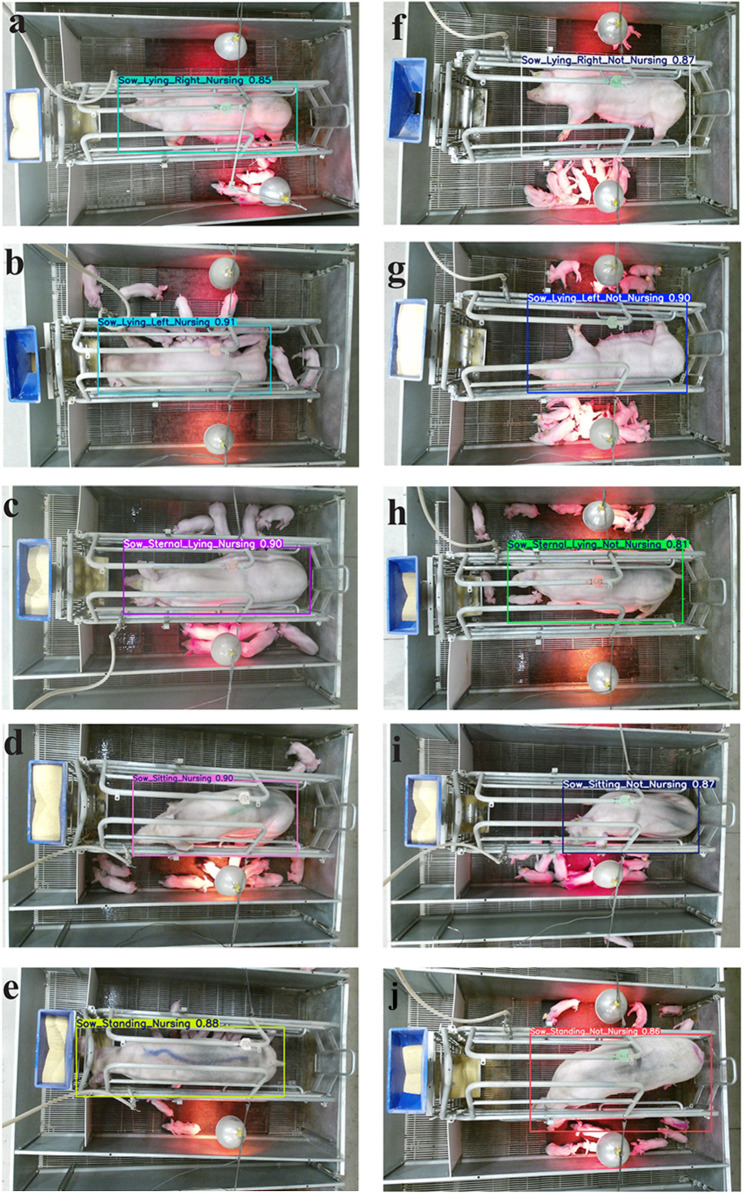
Predictions on test images via the modified YOLO11n model. Panels (**a–e**) show example nursing-labeled predictions by the model: (**a**) Sow_Lying_Right_Nursing, (**b**) Sow_Lying_Left_Nursing, (**c**) Sow_Sternal_Lying_Nursing, (**d**) Sow_Sitting_Nursing, and (**e**) Sow_Standing_Nursing. Panels (**f–j**) show not-nursing-labeled predictions by the model: (**f**) Sow_Lying_Right_Not_Nursing, (**g**) Sow_Lying_Left_Not_Nursing, (**h**) Sow_Sternal_Lying_Not_Nursing, (**i**) Sow_Sitting_Not_Nursing, and (**j**) Sow_Standing_Not_Nursing. The bounding boxes highlight clear posture detection with confidence scores, illustrating posture localization and example confidence scores under varying environmental conditions

The qualitative performance of the modified YOLO11n architecture was evaluated via predictions on test images, as shown in Fig. 7. The results indicate that the model detected and classified posture–nursing-state classes on the held-out test images, including frames with variable lighting and partial occlusion. Nursing-labeled classes, such as Sow_Lying_Left_Nursing and Sow_Sternal_Lying_Nursing, were predicted with confidence scores often exceeding 0.85 in these examples, suggesting stable outputs for these classes in the evaluated images.

Not-nursing-labeled classes and nursing-labeled classes were detected with high overall performance; the most frequent confusion occurred between Sow_Sitting_Nursing and Sow_Sitting_Not_Nursing, consistent with limited visibility of piglet-to-teat contact from the top-view angle. This is consistent with earlier findings from the confusion matrices, which highlighted higher misclassification rates for these ambiguous classes.

The bounding boxes generated by the model were well aligned with the sows’ postures, even under occlusions caused by piglets or varying lighting conditions. These qualitative results support the feasibility of real-time, frame-level posture–nursing-state classification in the evaluated setting; broader on-farm performance and generalization require external validation.

## Discussion

### Architectural modifications and efficiency gains

This study highlights the potential of a modified YOLO11n model, optimized using TensorRT, to enable real-time monitoring of posture–nursing-state of sows inside farrowing crates through an efficient computational platform. The removal of the small-object detection head improved computational efficiency while maintaining comparable mAP@50 (with lower mAP@50:95). The model targeted the experimental needs of sow behavior monitoring by ignoring small-object detection since this process was unnecessary for detecting and classifying a sow’s posture–nursing-state given the sow’s moderate to large body size.

Hidayatullah et al. developed the DeepSperm model, where a single detection layer was explicitly used for small-object detection in densely populated bull sperm cells [37]. Refining the architecture and eliminating redundant detect heads, they achieved a 2.18× speedup in inference and a 2.9× faster training time compared to YOLOv4 while maintaining high detection accuracy. These findings are consistent with the results obtained in this study, where an architectural alteration to YOLO11n simplified the model and yielded inference times of 4.6 ms on NVIDIA A100 and 6.1 ms on T4 GPUs after TensorRT optimization, supporting task-specific architectural simplification as a practical pathway to reduce latency.

Beyond inference speed, the qualitative performance of the modified YOLO11n model was evaluated on unseen test images, supporting its ability in identifying nursing-state–labeled and not-nursing-state–labeled classes under varying conditions, including occlusions and variable lighting. This observation is consistent with Zhou et al., who utilized YOLOv8 with an inspection robot to track lactating sows and piglets, demonstrating a 97.08% mAP with TensorRT acceleration [38]. Their results suggest that optimizing object detection models for specific tasks, whether through architectural simplifications or targeted optimizations, can yield substantial efficiency gains while supporting reliable classification under the evaluated conditions.

Furthermore, Yang et al. proposed a deep learning-based segmentation approach for sow nursing behavior recognition that integrated spatial and temporal features to improve detection accuracy in commercial farm conditions [39]. Their methodology demonstrated 96.4% accuracy in nursing behavior recognition, emphasizing the importance of combining architectural efficiency with domain-specific modifications. In the same vein, this study indicates that the targeted removal of detection heads and optimized model structure can yield significant efficiency improvements, making real-time livestock behavior monitoring more feasible in farm environments.

### Performance and computational trade-offs

The modified YOLO11n achieved an mAP@50 of 98.90%, comparable to larger models such as YOLO11 × (98.79%) and YOLO11l (98.85%) while maintaining a significantly smaller model size and reduced computational complexity. Despite its lightweight architecture, YOLO11n preserved high detection accuracy across all ten posture–nursing-state classes, demonstrating strong efficiency in precision livestock farming (PLF) applications. Notably, the model achieved an inference time of 4.6 ms on the NVIDIA A100 GPU and 6.1 ms on the T4 GPU when optimized with TensorRT, making it well-suited for real-time behavior monitoring in livestock facilities.

The trade-off between detection accuracy and computational efficiency is critical for deploying deep learning models in real-world PLF environments. Larger models, such as YOLO11x, demonstrated a slightly higher mAP@50:95 (94.23%), but their significantly higher inference times (total inference time on the T4: 27.4 ms after TensorRT) limit their suitability for low-latency edge computing scenarios. This observation is consistent with findings from Tsai et al., in which a YOLOv7-based tracking model for piglet monitoring achieved high accuracy (94.6%) but required substantial computational resources, limiting its real-time applicability in embedded systems [24]. Additionally, prior research by Yang et al. emphasized the need for efficient architectures in sow behavior detection, particularly for systems constrained by power and hardware limitations [39]. The proposed YOLO11n_modified model effectively balances precision, speed, and computational efficiency, and the A100/T4 benchmarks indicate that the modified variant remains competitive when latency constraints are prioritized over mAP@50:95. Deployment feasibility on embedded devices (e.g., Jetson-class platforms) requires dedicated benchmarking that accounts for precision mode, input resolution, and end-to-end I/O, but results are promising regarding deployability.

### Classification challenges and misclassifications

Despite the strong overall accuracy of the modified YOLO11n model, certain posture–nursing-state classes presented classification difficulties, particularly those with subtle visual differences. Classification ambiguity was most pronounced when piglet-to-teat contact was partially occluded in top-view imagery, particularly during sitting or standing postures that can coincide with nursing termination; therefore, the nursing label in this study should be interpreted as visible udder/teat-line engagement rather than a complete nursing bout. Classes such as Sow_Sitting_Not_Nursing and Sow_Sitting_Nursing exhibited higher misclassification rates due to limited visibility from the top-view camera angle and minimal postural variations, emphasizing a key challenge in automated behavior recognition. In the annotation protocol, Sow_Sitting_Nursing was labeled when piglets were at the udder region with an apparent nursing attempt, whereas Sow_Sitting_Not_Nursing was assigned when no piglets were at the udder region or oriented toward nursing. Küster et al. encountered similar classification challenges, achieving an overall accuracy of only 59.6% in identifying sow postures and behaviors using an earlier version of YOLO, but their study classified general postures rather than nursing vs. not-nursing states [40]. Their study highlighted the inherent difficulty in differentiating behaviors with overlapping visual characteristics, even with a well-structured deterministic classification approach.

A detailed analysis of the confusion matrix further revealed that even larger models, such as YOLO11l, struggled to resolve these fine-grained distinctions, indicating that increased model complexity does not inherently translate to superior classification performance for closely related classes. This is consistent with previous research findings, which suggest that excessive architectural complexity can introduce additional computational overhead without necessarily enhancing discriminatory power [24].

However, the modified YOLO11n model improved class separability, particularly for distinct postural classes such as Sow_Lying_Left_Not_Nursing and the background class, supporting its potential applicability in PLF under the evaluated conditions. The optimization techniques, including TensorRT acceleration, helped maintain computational efficiency while preserving classification robustness.

The decreasing training and validation loss values are consistent with model convergence. The low loss values indicate close agreement between predictions and labels within the training/validation sets, while the residual confusion for sitting and sternal-lying pairs indicates that viewpoint-driven ambiguity remains a limiting factor for frame-level classification.

### Practical implications of TensorRT optimization

TensorRT optimization improved the practical implementation of the modified YOLO11n model by leveraging advanced techniques such as kernel fusion, mixed-precision calibration, and dynamic tensor memory allocation. The optimizations resulted in substantial reductions in inference latency, achieving speedups of 58.56% on the NVIDIA A100 GPU and 44.55% on the T4 GPU, supporting low-latency inference on the evaluated GPU platforms; deployment in commercial barns will depend on edge hardware benchmarks and end-to-end camera I/O constraints. The acceleration provided by TensorRT may enable high-speed inference on lower-power platforms, but accuracy and throughput should be re-validated after conversion and deployment-specific optimization, which is essential for the large-scale deployment of deep learning-based livestock monitoring solutions.

The modified YOLO11n requires 5.0 GFLOPs, which makes it a plausible candidate for low-cost edge devices such as NVIDIA Jetson Nano based on reported peak theoretical compute throughput [41]. This comparison provides a coarse upper-bound feasibility check; realized throughput on embedded platforms will also depend on memory bandwidth, precision mode (FP16/INT8), image resolution, and end-to-end I/O latency.

Results from Ma et al. demonstrate similar advantages of TensorRT in unmanned aerial vehicle (UAV) applications that showcase technology-enabled real-time object detection and reduced computational overhead on embedded systems [34]. These findings are consistent with the presented results, which show that lightweight, TensorRT-optimized models can significantly improve inference speeds while maintaining high detection accuracy, making them suitable for continuous monitoring tasks in dynamic farm environments. Furthermore, the study by Vinoth & Sasikumar on low-light object detection reinforced that TensorRT accelerates inference and enhances robustness in challenging scenarios, such as variable lighting and occlusion [35]. Collectively, these studies suggest that TensorRT can reduce inference latency across several application domains; in our setting, the measured speedups on A100 and T4 support feasibility for low-latency deployment under the evaluated conditions.

In addition, Wang’s research highlighted TensorRT’s effectiveness in lowering energy consumption and reducing inference time, displaying a significant reduction in GPU power usage while maintaining high detection accuracy with a 50 ms reduction in inference time [36]. This is particularly relevant in large-scale agricultural operations, where power-efficient solutions are essential for cost-effective, long-term monitoring.

### Limitations

The nursing-state labels in this study reflect an operational proxy based on top-view, frame-level visibility of piglet snout-to-udder/teat-line contact and do not confirm milk letdown, milk transfer, or the onset and termination of complete nursing bouts. Occlusions and camera perspective can hide piglets at the udder and introduce label noise, which likely contributes to residual confusion between visually similar posture–nursing-state pairs (notably sitting nursing vs. sitting not nursing). The dataset originates from a single facility and a limited set of stall layouts, and the train–validation split was performed at the image level; external validation across herds, facilities, and camera placements is required to estimate generalization. The reported inference times reflect model inference under the evaluated settings and do not represent end-to-end system latency (e.g., video decoding, data transfer, and on-farm networking). Edge-device feasibility is discussed using theoretical throughput rather than direct benchmarking and should be interpreted as indicative only.

## Conclusions

This study highlights the potential of the modified YOLO11n model optimized with TensorRT for low-latency, frame-level monitoring of sow posture–nursing-state classes in farrowing crates. The model achieved an mAP@50 of 98.90%, with inference speeds of 4.6 ms on the NVIDIA A100 GPU and 6.1 ms on the T4 GPU, demonstrating a favorable trade-off between detection accuracy and computational efficiency. Key architectural modifications, such as removing the small-object detection head, reduced the model’s complexity while maintaining strong detection performance on the evaluated dataset. TensorRT optimization increased the inference speed, reducing the latency by up to 58.56% and supporting deployment across the evaluated GPU platforms. While the model faces challenges distinguishing visually similar posture–nursing-state classes, the limitations are driven by the operational label definition and viewpoint constraints. In this study, “nursing” labels reflect visually confirmable piglet snout/mouth contact toward the udder/teat line in top-view frames and do not confirm milk transfer or complete nursing bouts, particularly when piglets are occluded beneath the sow or when contact is not visible. The evaluation is frame-level and based on data from the studied facility and camera configuration, so generalization to other barns, lighting conditions, crate designs, and camera placements requires external validation. Future research should address these limitations by expanding datasets, benchmarking end-to-end performance on edge hardware, and adding lightweight temporal post-processing to reduce frame-to-frame label flicker.

These results indicate that the proposed approach provides a computationally efficient, low-latency framework for PLF. The model can support welfare-oriented monitoring workflows through automated posture-nursing-state classification, which may help inform timely management decisions in modern swine production systems.

Future work will evaluate lightweight temporal post-processing (e.g., sliding-window majority voting or an exponential moving average of class probabilities) to reduce frame-to-frame label flicker in continuous video streams and improve on-farm deployment stability.

## Data Availability

No datasets were generated or analysed during the current study.
